# The anti-staphylococcal fusidic acid as an efflux pump inhibitor combined with fluconazole against vaginal candidiasis in mouse model

**DOI:** 10.1186/s12866-024-03181-z

**Published:** 2024-02-10

**Authors:** Salwa E. Gomaa, Hisham A. Abbas, Fatma A. Mohamed, Mohamed A. M. Ali, Tarek M. Ibrahim, Alyaa S. Abdel Halim, Mashael A. Alghamdi, Basem Mansour, Anis Ahmad Chaudhary, Amr Elkelish, Fehmi Boufahja, Wael A. H. Hegazy, Fatma Al-zahraa A. Yehia

**Affiliations:** 1 Department of Microbiology and Immunology, Faculty of Pharmacy, Zagazig University, Zagazig, 44519 Egypt; 2 Department of Medical Microbiology and Immunology-Medical School, University of Pécs, Szigeti Út 12, Pécs, H-7624 Hungary; 3 Department of Biology, College of Science, Imam Mohammad Ibn Saud Islamic University (IMSIU), Riyadh, 11623 Saudi Arabia; 4 Department of Biochemistry, Faculty of Science, Ain Shams University, Abbassia, Cairo, 11566 Egypt; 5 Department of Pharmaceutics, Faculty of Pharmacy, Zagazig University, Zagazig, 44519 Egypt; 6 Department of Chemistry, Imam Mohammad Ibn Saud Islamic University (IMSIU), Riyadh, 11623 Saudi Arabia; 7 Department of Pharmaceutical Chemistry, Faculty of Pharmacy, Delta University for Science and Technology, Gamasa, Belqas 11152 Egypt; 8 Department of Botany and Microbiology, Faculty of Science, Suez Canal University, Ismailia, 41522 Egypt; 9 Pharmacy Program, Department of Pharmaceutical Sciences, Oman College of Health Sciences, Muscat, 113 Oman

**Keywords:** Drug repurposing, Vaginal candidiasis, Fluconazole resistant *Candida albicans*, Fusidic acid, Efflux pumps

## Abstract

**Background:**

*Candida albicans* is the most common fungus that causes vaginal candidiasis in immunocompetent women and catastrophic infections in immunocompromised patients. The treatment of such infections is hindered due to the increasing emergence of resistance to azoles in *C. albicans*. New treatment approaches are needed to combat candidiasis especially in the dwindled supply of new effective and safe antifungals. The resistance to azoles is mainly attributed to export of azoles outside the cells by means of the efflux pump that confers cross resistance to all azoles including fluconazole (FLC).

**Objectives:**

This study aimed to investigate the possible efflux pump inhibiting activity of fusidic acid (FA) in *C. albicans* resistant isolates and the potential use of Fusidic acid in combination with fluconazole to potentiate the antifungal activity of fluconazole to restore its activity in the resistant *C. albicans* isolates.

**Methods:**

The resistance of *C. albicans* isolates was assessed by determination of minimum inhibitory concentration. The effect of Fusidic acid at sub-inhibitory concentration on efflux activity was assayed by rhodamine 6G efflux assay and intracellular accumulation. Mice model studies were conducted to evaluate the anti-efflux activity of Fusidic acid and its synergistic effects in combination with fluconazole. Impact of Fusidic acid on ergosterol biosynthesis was quantified. The synergy of fluconazole when combined with Fusidic acid was investigated by determination of minimum inhibitory concentration. The cytotoxicity of Fusidic acid was tested against erythrocytes. The effect of Fusidic acid on efflux pumps was tested at the molecular level by real-time PCR and in silico study. In vivo vulvovaginitis mice model was used to confirm the activity of the combination in treating vulvovaginal candidiasis.

**Results:**

Fusidic acid showed efflux inhibiting activity as it increased the accumulation of rhodamine 6G, a substrate for ABC-efflux transporter, and decreased its efflux in *C. albicans* cells. The antifungal activity of fluconazole was synergized when combined with Fusidic acid. Fusidic acid exerted only minimal cytotoxicity on human erythrocytes indicating its safety. The FA efflux inhibitory activity could be owed to its ability to interfere with efflux protein transporters as revealed by docking studies and downregulation of the efflux-encoding genes of both ABC transporters and MFS superfamily. Moreover, in vivo mice model showed that using fluconazole-fusidic acid combination by vaginal route enhanced fluconazole antifungal activity as shown by lowered fungal burden and a negligible histopathological change in vaginal tissue.

**Conclusion:**

The current findings highlight FA’s potential as a potential adjuvant to FLC in the treatment of vulvovaginal candidiasis.

## Background

Candidiasis is a worldwide fungal infection that is caused by a wide range of *Candida* species. Most cases of candidiasis are caused by the yeast *Candida albicans*, which can be invasive and has a high recurrence rate, especially in patients with impaired immunity [1, 2].

Mucosal infections are also widespread, and vulvovaginal candidiasis (VVC) is being considered the second most common cause of vaginal infection [3]. This is due to the elevated vaginal epithelium content of glycogen caused by the increased estrogen levels, creating an ideal condition for the growth of fungi [4]. Vulvovaginal candidiasis is predominantly caused by *C. albicans* (in about 90% of cases). About 70% of all women are at risk of having at least one episode of VVC during their fertile years [4]. The most vulnerable are women who are at childbearing age. VVC manifests as vaginal itching, burning, redness, and increased vaginal discharge [5].

Due to inappropriate treatment, prolonged or recurrent illness may result in recurrent vulvovaginal candidiasis [5].

Common antifungals are available for VVC including azoles, polyenes, and echinocandins [6, 7]. Azoles are widely used in clinics and are considered as a first-line agent against candidiasis. Azoles act by inhibiting 14-α-demethylase that is encoded by *ERG11* gene and plays a crucial role in the biosynthesis of ergosterol, resulting in decreasing ergosterol levels in the fungal cell membrane [8–10]. Among azoles, fluconazole (FLC) is the most commonly used for VVC prophylaxis and treatment. However, its widespread clinical use has led to the emergence of FLC-resistant strains, treatment failures, and recurring infections [8].

Several mechanisms contribute to azole resistance in *Candida* such as overexpression or mutations in *ERG11* and up-regulation of efflux pumps [11, 12]. Among the most frequent azole resistance mechanism is the upregulation of membrane transporters [8, 13]. The overexpression of ABC transporters (CDR1) encoded by *CDR1* gene and (CDR2) encoded by *CDR2* gene, in addition to the MFS transporters (Mdr1) encoded by *MDR1* gene greatly contributes to *C. albicans* resistance to FLC [14]. It was reported that the downregulation of *CDR1*, *CDR2*, and *MDR1* genes’ expression has increased *C. albicans* susceptibility to FLC [15].

Using azoles in conjunction with other non-antifungals is a promising strategy against resistant *C. albicans* infections. Many of these combinations have synergistic effects against resistant *C. albicans* strains [16]. This synergism may be due to various mechanisms, including decreasing antifungal drug efflux via suppressing the expression of efflux pump genes, increasing fungal membrane permeability by inhibiting sterol synthesis, biofilm inhibition, inhibiting the activity of enzymes and proteins essential for the survival of fungal cell, and disrupting intracellular ion homeostasis [16–18].

Drug repurposing is a novel approach that could be beneficial in minimizing the high economic cost and the time of developing new antifungals [19]. Verapamil was reported to inhibit the efflux pumps in FLC-resistant *C. albicans* as well as exhibited synergistic effect with FLC [20]. Rhodamine 6G (R6G) and rhodamine-123 (R123) have been identified as fluorescent substrates of fungal efflux pumps and are therefore used for assaying the activity of novel efflux pump inhibitors (EPI) [21]. In silico techniques were employed in a few studies to identify and validate EPI [22]. Molecular docking is one of the in silico approaches that has been recently utilized to investigate molecules capable of binding to efflux pump proteins in *Candida* spp. [23, 24].

Fusidic acid (FA) is an antibacterial that was originally isolated from the fungus named *Fusidium coccineum.* FA is a commercially marketed anti-staphylococcal drug that is available in oral, parenteral, and topical (ointments and creams) formulations. FA acts by inhibiting protein synthesis through inhibition of elongation factor G [25–27]. It was reported that FA and its derivatives have broad spectrum pharmacological activities such as antibacterial, antifungal, antituberculosis, antiparasitic, anticancer, anti-inflammation, and antiviral activity in vivo and in vitro [28]. This study aimed to investigate the possible efflux pump inhibiting activity of fusidic acid in *C. albicans* resistant isolates and the potential use of fusidic acid in combination with fluconazole to potentiate the antifungal activity of fluconazole to restore its activity in the resistant *C. albicans* isolates.

## Methods

### Fungal strains

Five FLC-resistant *C. albicans* isolates (CA3, CA13, CA24, CA29, and CA46) were employed in this study. The isolates were obtained from patients admitted to Zagazig University Hospitals, Egypt, and initially identified biochemically with further confirmation using chromogenic agar. All strains were kept in Yeast Peptone Dextrose (YPD) broth supplemented with 15% glycerol (vol/vol) at − 80 °C and freshly subcultured on YPD agar plates prior to each experiment.

### Minimum Inhibitory Concentrations (MICs) determination

Due to solubility problems, the agar dilution method was used to determine the MICs of FLC and FA (Sigma, St. Louis, USA) [29, 30]. Briefly, sterile molten Sabouraud Dextrose Agar (SDA) (45–50 °C) was mixed with previously prepared varied concentrations of FA (16,000- 125 µg/mL) or FLC (8192- 64 µg/mL), thoroughly mixed, and placed into sterile petri dishes, so that the tested agents existed in varying concentrations on each plate. The agar plates were left at room temperature to solidify and the agar surface was dried in a laminar flow hood for 20 min before inoculating.

Overnight of *C. albicans* cultures were diluted using sterile saline to match 0.5 McFarland Standard (1–5 × 10^6^ CFU/mL) and then were 1:10 diluted using sterile saline. Aliquots of 2 µL of the diluted suspensions were inoculated onto the surface of the prepared SDA plates. Plates devoid of any drugs were utilized as a growth control. After incubation at 37 °C for 16–20 h, the lowest concentrations of the FLC and FA that prevent *Candida* visible growth were considered as the MICs values.

### The effect of sub-inhibitory fusidic acid concentrations on *Candida* viability

The effect of sub-MIC of FA on *C. albicans* growth was assessed [31, 32]. All strains were grown in YPD broth with and without sub-MIC of FA (1000 µg/mL) and incubated overnight at 37 °C. A spectrofluorometer (Biotek, USA) was used to compare the optical densities (OD) of *Candida* cultures with and without FA addition at 600 nm in addition to viable counts to assure that this concentration which would be used in further experiments had no effect on fungal growth.

### Assay of rhodamine 6G (R6G) efflux

An efflux assay using R6G was done to determine whether FA affects efflux pump activity according to Liu X et al.*,* with some adjustments [33]. Briefly, log phase cultures of *C. albicans* were done in YPD broth from overnight ones. Cultures were centrifuged and the pellet was rinsed three times with phosphate-buffered saline (PBS), resuspended to a final concentration (1 × 10^8^ cells/mL) in glucose-free PBS, and left for 2 h at 30 °C to induce starvation. Then, R6G (10 μM final concentration) was added followed by incubation for 30 min to allow rhodamine accumulation under energy-depleting conditions. The extracellular R6G was removed by harvesting the cell suspensions and washing them three times in glucose-free PBS. FA (1000 μg/mL) was added to the cell suspensions. Rhodamine efflux was induced by adding glucose (10 mM final concentration) and incubating for 20 min. Cell suspensions were centrifuged and the fluorescence intensity of the effluxed rhodamine was recorded in the supernatants at an excitation wavelength (529 nm) and an emission wavelength (553 nm) using a spectrofluorometer (Biotek, USA). The fluorescence intensity was expressed in arbitrary units (a.u.).

### Rhodamine 6G intracellular accumulation

R6G intracellular accumulation was assessed as previously mentioned with some modifications [34]. Briefly, the R6G-loaded cells were treated with FA (1000 μg/mL) followed by glucose addition (10 mM final concentration) and incubation for 20 min as previously mentioned. The cells were then collected by centrifugation at 5000 × g for 5 min at 4 °C before being rinsed twice with cold PBS. The resulting pellet was examined using a Leica DM500 fluorescence microscope, Leica, Germany. The Image J software was used to measure the fluorescence intensity.

### Quantification of ergosterol content

FA was tested for its effect on ergosterol biosynthesis in *C. albicans* as described previously with some modifications [23]. In brief, *C. albicans* isolate (CA29) was grown in YPD broth with and without FA (1000 μg/mL) and incubated at 35 °C with shaking for 16 h. Afterthat, *Candida* cultures were centrifuged at 2,700 rpm for 5 min, the resulting cell pellets were rinsed once. Aliquots of 3 mL of alcoholic solution of potassium hydroxide (25%) was added to each cell pellet and mixed by vortexing for 1 min. The cell suspensions were then transferred into sterile screw-capped borosilicate glass tubes and incubated in a water bath at 85 °C for 1 h. Following incubation, the tubes were left to cool at room temperature.

To extract sterols, aliquots of 3 mL of *n*-heptane and 1 mL of sterile water were added, mixed by vigorous vortexing for 5 min then allowed to stand for 15 min. The heptane layer containing total sterols was transferred to a clean borosilicate glass tube with a screw cap and stored for as long as 24 h at -20 °C. Before analysis, 1 mL aliquot of the heptane layer was fivefold diluted with 100% ethanol before scanning with UV–visible spectrophotometer (6800 Double Beam Spectrophotometer) between 230 and 300 nm. The absorbances of the extracted sterols in samples resulted in a four-peak curve. Both ergosterol and 24(28)-dehydroergosterol absorb at 281·5 nm, however, only the latter absorbs at 230 nm. The percentage of ergosterol in FA-treated culture compared to that in untreated control was calculated.

### Drug potentiation

To determine FLC effectiveness in the presence of FA, the MIC of FLC was reassessed in the presence of FA's sub-MIC using the broth micro-dilution method [23]. Two-fold serial dilutions of FLC were performed in Sabouraud Dextrose Broth (SDB) in the wells of microtitre plates. FA was dispensed into all wells at a sub-MIC value. After that, 1:100 diluted *C. albicans* isolates were added to all wells. Following 48 h of incubation, MIC_80_ is defined as the lowest concentration at which growth is reduced by 80%.

### Assay of hemolytic activity

The hemolytic activity of FA was assessed as described previously with some modifications [35]. Briefly, freshly collected human red blood cells (hRBCs) in an anti-coagulant were washed three times with PBS then suspended in PBS (4% v/v). FA (1000 μg/mL) was added to the prepared RBCs suspension at a final volume of 1 mL and the mixture was incubated for 35 min at 37 °C. The hemoglobin release was monitored after centrifuging the sample at 2000 rpm for 2 min and the absorbance of the supernatant was determined at 540 nm (A_sample_). Positive and negative controls were the hRBCs in 1% (v/v) Triton X-100 (A_Triton_) and in PBS (A_blank_), respectively. The following formula was used to calculate the percent (%) of hemolysis:

$${\text{Hemolysis}}\left(\%\right)={\left[\left({A}_{sample}-{A}_{blank}\right)/\left({A}_{Triton}-{A}_{blank}\right)\right]}^{*}100.$$

### qRT-PCR

The effect of FA on the expression level of *CDR1*, *CDR2,* and *MDR1* in the tested *C. albicans* isolate (CA29) was assayed by qRT-PCR. *C. albicans* isolate was grown in YPD broth with and without FA (1000 μg/mL) and incubated overnight at 30 °C. After centrifugation, TRIzol Reagent (15596026, Life Technologies, USA) was used to extract and purify the total RNA from *Candida* isolate following the manufacturer instructions. The cDNA was synthetized using the QuantiTect Reverse Transcription Kit. The amplification of the cDNA product was done using Thermo Scientifc Maximas SYBR Green/Fluorescein qPCR Master Mix. The sequence of primers used is shown in Table 1. The relative expression level of the tested genes was normalized to the house-keeping gene (*ACT1*) using the 2^−ΔΔCt^ method [36–38]. The experiment was done in triplicate.

**Table 1:** Primers used in this study

Gene name	Primer sequence	Reference
*CDR1*	F/5′-GGAGTTTGGGTGCTGTTTGT-3′ R/5′-AATTCAACCCCAATGGTCAA-3′	[23]
*CDR2*	F/5′-AAAAAGGTGGAAGAACGGC-3′ R/5′-TTGGCATGAGATCCTGGTG-3′	[39]
*MDR1*	F/5′-GGAGTTTGGGTGCTGTTTGT-3′ R/5′-TGTGGTACCCAATTCAACGA-3′	[23]
*ACT1*	F/5′-TTTTGACCTTGAGATACCCA-3′ R/5′-GGAGCTCTGAATCTTTCGTT-3′	[23]

### In silico study

For the docking study was conducted as describerd earlier [40], FA was drawn in the Marvin Sketch of Marvin suite (http://www.chemaxon.com) and the most stable conformer for FA was generated. The 3D structure of UniProtKB – Q5ABU7 (MDR1_CANAL) Multidrug resistance protein 1 of the efflux pump encoded by *MDR1* gene [41, 42], UniProtKB—Q5ANA3 (CDR1_CANAL) Pleiotropic ABC efflux transporter of multiple drugs CDR1 encoded by *CDR1* gene [43, 44] and UniProtKB -P78595 (CDR2_CANAL) Multidrug resistance protein CDR2 encoded by *CDR2* gene, all are expressed normally in *C. albicans* [44, 45] and were drawn in PDB format as AlphaFold predicted structures (https://alphafold.ebi.ac.uk/).

### Preparation of vaginal gels

The vaginal gels were prepared by incorporation of FLC, FA, or a mixture of both pre-mentioned drugs into Carbopol 940 as a hydrogel base. Firstly, the drugs were individually dissolved in absolute ethanol (0.25 mL) and tween 80 (0.25 mL) using a magnetic stirrer. Then, distilled water was added portionwise until the final volumes reached 5 mL. Carbopol 940 powder (0.4% w/v) was spread on the liquid preparations with continuous stirring until homogeneous gels were formed. To be suitable for vaginal application, the gels were targeted to have a pH of 4–4.5 by addition of triethanolamine drops [46]. Per 5 g of each gel preparation, 1.8 mg FLC, 5 mg FA, or 1.8 mg FLC-5 mg FA mixture were used. The gels were kept in the refrigerator overnight for complete gel dispersion.

### Vaginal candidiasis in vivo model

The effect of the prepared gels on the pathogenicity of *C. albicans* isolate (CA29) was assessed using a VVC mouse model. The experiment was designed according to Muñoz et al*.* [47]*,* and Qu et al*.,* [48, 49]. Thirty (18–20 g) female albino mice were obtained from the animal house in the Faculty of Pharmacy, Zagaig University (approval for in vivo experiments usage ZU-IACUC/3/F/399/2022). Mice were rendered immunocompromised by being intraperitoneally injected with cyclophosphamide daily for 3 successive days (100 mg/kg body weight). In the meantime, mice were subcutaneously injected with 0.2 mg of estradiol benzoate dissolved in sesame oil for 3 successive days to induce the pseudo-oestrus phase before vaginal inoculation. Mice were then anesthetized with urethane (1.25 mg/kg) and inoculated with 6 × 10^8^ CFU/mL CA29 spores (20 μL/mice) into the vaginal lumen. One day post infection, mice were randomly selected, and their vaginas were lavaged with sterile PBS, serially diluted, and cultured on YPD agar for 48 h at 30 °C for colony count to assure *Candida* infection.

Mice were divided randomly into 6 groups of 5 mice each; in the 1^st^ group, mice were left uninfected as a negative-control group, in the 2^nd^ group, mice received ethanol gel daily as a vehicle control group, and the other groups, mice were infected. The infected groups were as follows; in the 3^rd^ group, mice were left untreated as a positive-control. The 4^th^ group was given FLC gel, and the 5^th^ group was given FA gel (1000 μg/mL). Mice in the 6^th^ group received [FA (1000 μg/mL)-FLC] gel. Mice received the vaginal gel preparations (20 μL/mice) once daily for 5 days. The fundal burden in lavage was assessed after 5 days and mice were sacrificed and their vaginas were collected for histopathological analysis.

### Statistical analysis

Three independent replicates of each experiment were conducted. The data was presented as mean ± standard error. Otherwise mentioned, analysis was done using a student's t test. *P* < 0.05 was regarded as statistically significant.

## Results

### Fusidic acid did not interfere with *Candida* viability

**Fig. 1:**
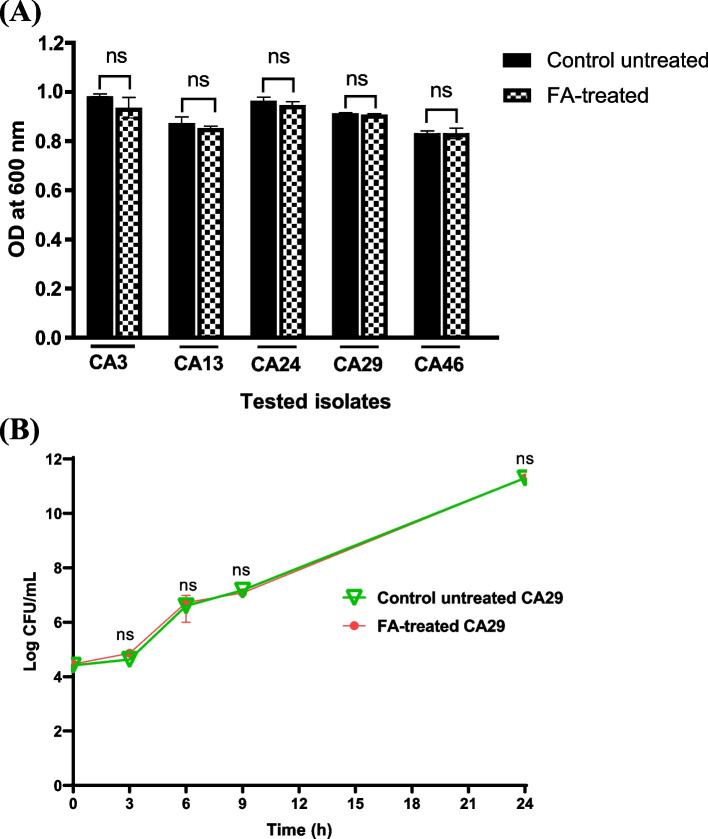
*C. albicans* viability was unaffected by FA at sub-MIC value of 1000 μg/mL. **A** No significant inhibition was observed in the growth rates of either FA-treated or untreated cultures at OD 600 nm. **B** Time kill assay was performed to a representative strain, there was no significant difference between the *C. albicans* growth at any time point. Data displayed was mean ± standard error. ns; non-significant (*P* > 0.05)

FA had MICs values of > 16000 μg/mL against all tested *Candida* isolates. The sub-MIC (1000 μg/mL) of FA was chosen for further experiments. No significant effect was observed in the OD of both treated and untreated cultures, indicating that 1000 μg/mL did not affect *Candida* viability (Fig. 1).

### Fusidic acid inhibited extracellular rhodamine 6G efflux among FLC- resistant *C. albicans*

**Fig. 2:**
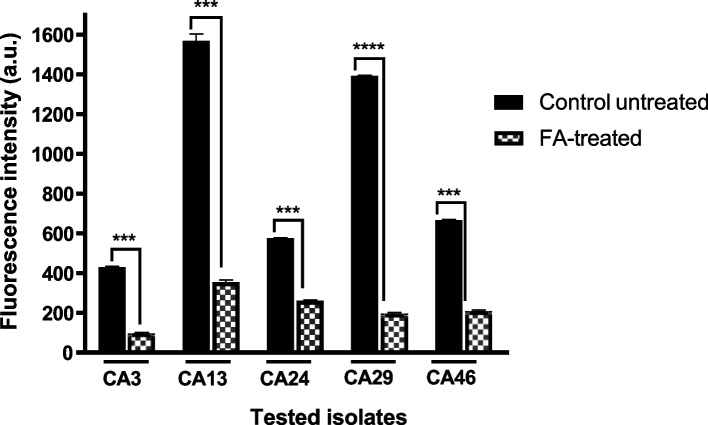
Reduced R6G efflux into the extracellular environment in FA-treated *C. albicans* (1000 μg/mL). The supernatants of FA-treated cells showed lower fluorescence intensity than that of the control untreated ones, 20 min following efflux initiation by addition of glucose. Fluorescence intensity was expressed in arbitrary units (a.u.). Data displayed was mean ± standard error. ***: *P* < 0.001

To investigate whether FA (1000 μg/mL) could affect *Candida* efflux pump transporters, the R6G efflux experiment was conducted. The fluorescence intensity of the extracellular R6G was measured in the supernatants of treated and untreated cells after 20 min of adding glucose. FA resulted in significant inhibition in the percent of emitted fluorescence (54.54%- 85.84%) among the tested isolates (Fig. 2). These data suggest the potential ability of FA to interfere with the efflux pump mediated transporters in *C. albicans*.

### Fusidic acid inhibited R6G extrusion

**Fig. 3:**
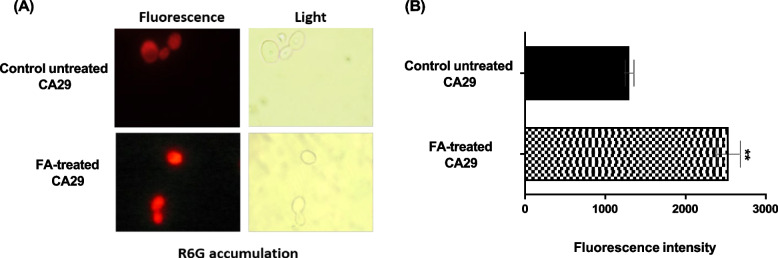
Accumulation of R6G inside FA-treated and untreated FLC-resistant *Candida* cells (1000 μg/mL). **A** Both bright field and fluorescence microscopy were used to observe R6G accumulation. FA-treated CA29 cells were able to retain more R6G intracellularly which is reflected by the intense fluorescence within the cells compared to the control untreated cells. **B** The Image J-calculated fluorescence intensity is shown in the bar graph. **: *P* < 0.01

To determine the activity of FA (1000 μg/mL) against *C. albicans* efflux pumps, FA-treated CA29 cells were examined for the buildup of a fluorescent dye (R6G). The fluorescence imaging showed higher fluorescence in the FA-treated cells, indicating more R6G dye accumulation as compared to the control untreated ones **(**Fig. 3A). The Image J software was used to measure the fluorescence intensity (Fig. 3B).

### Fusidic acid altered ergosterol level in fluconazole resistant *C. albicans*

**Fig. 4:**
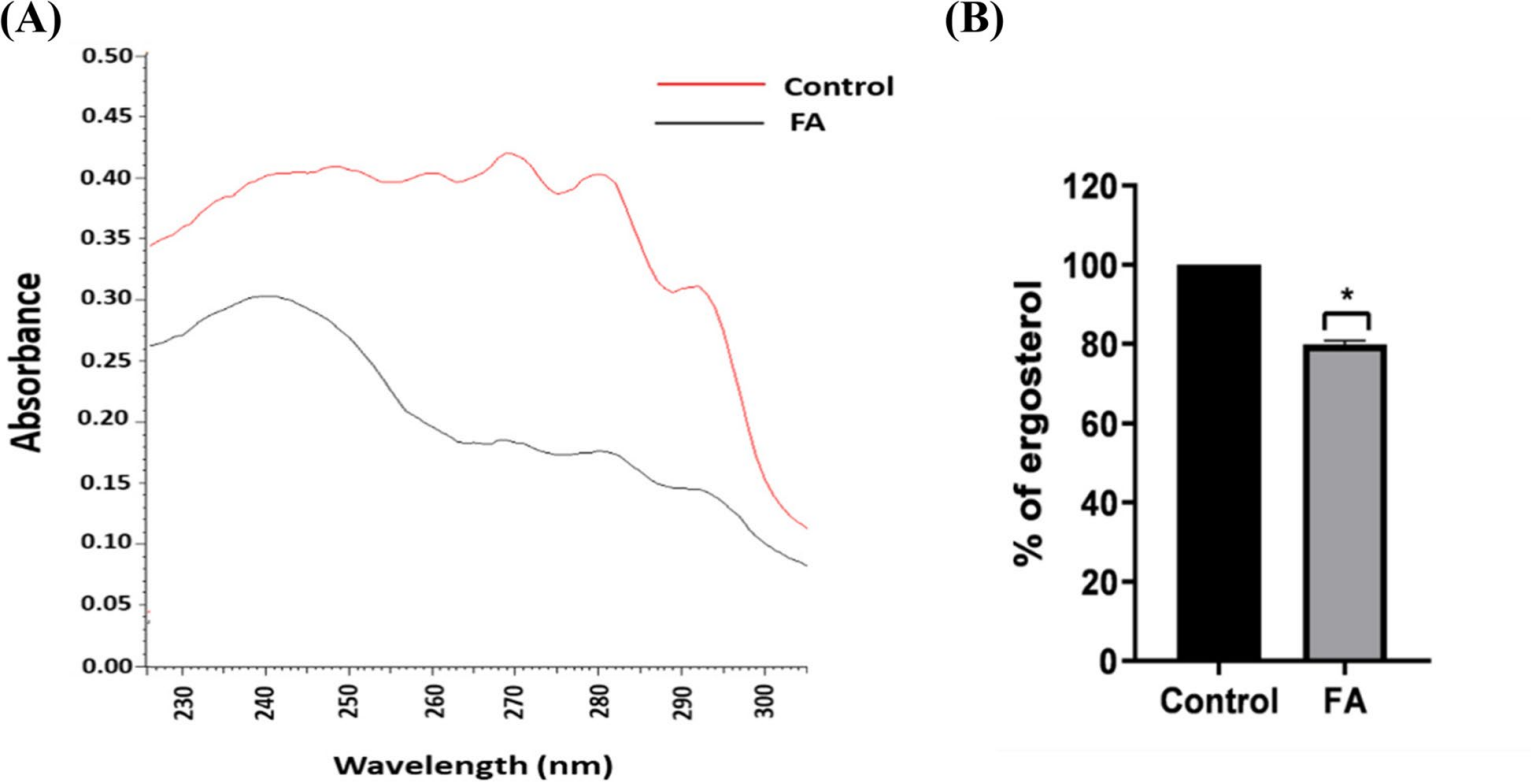
Reduced ergosterol content in *C. albicans* treated with FA (1000 μg/mL). **A** Ergosterol profile of FA-treated CA29 isolate (black line) and the control untreated one (red line) scanned with UV spectrophotometer between 230 and 300 nm. **B** Ergosterol level of FA-treated and control untreated CA29 isolate, expressed as a percentage. Data displayed was mean ± standard error. *: *P* < 0.05

The ergosterol content was analyzed to further explore whether FA has antifungal activity on *Candida* cell membrane. Treatment of FLC-resistant CA29 isolate with 1000 μg/mL FA, significantly decreased the amount of ergosterol by approximately 19.1% compared to the control untreated one. This may contribute to reversal of FLC-resistance in *C. albicans* because ergosterol is the primary target for FLC. Scans taken from both the control and FA-exposed CA29 isolate as well as the percent of ergosterol levels are shown in Fig. 4.

### Fusidic acid potentiated antifungal effect of fluconazole against *C. albicans*

**Fig. 5:**
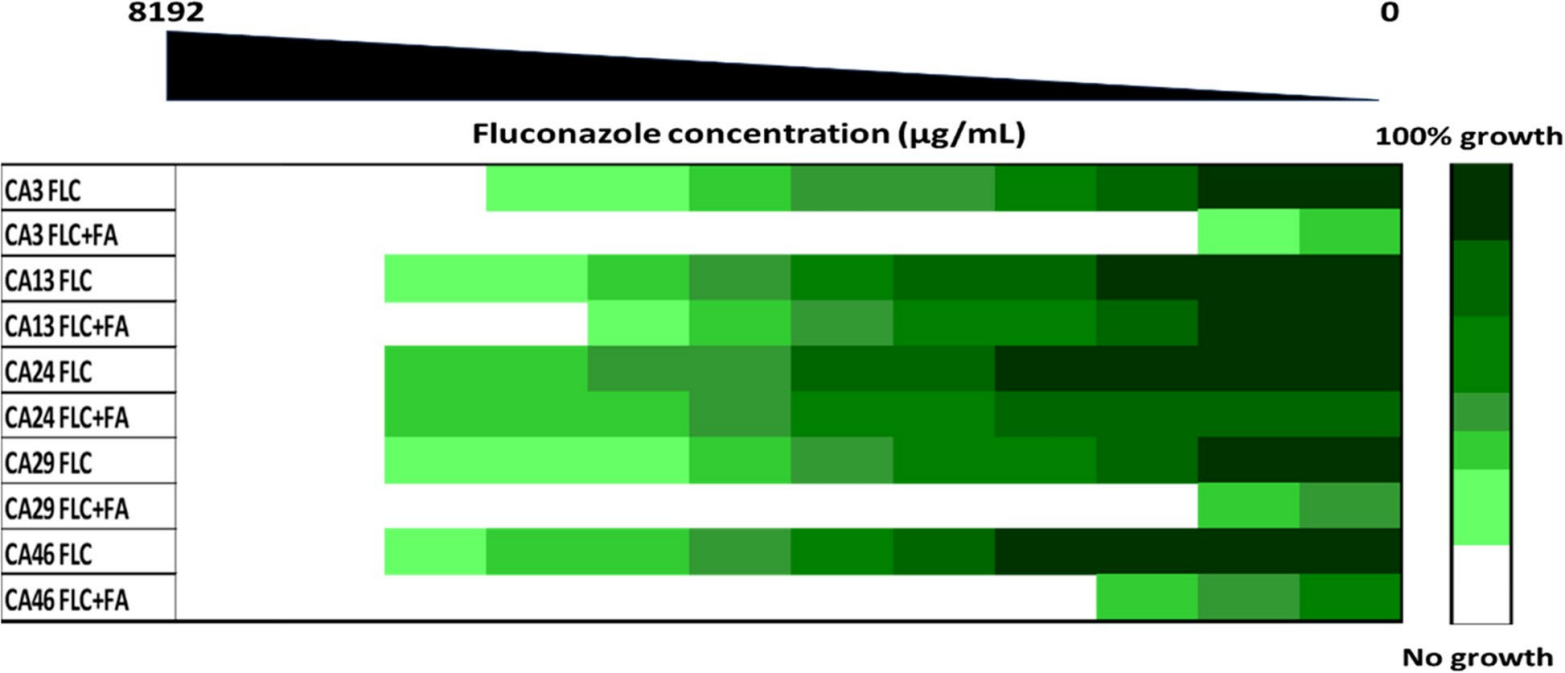
The antifungal activity of FLC against the tested *C. albicans* isolates was enhanced by the addition of FA (1000 μg/mL). The obtained data were plotted as a heat map

FA (1000 μg/mL) enhanced FLC efficiencies against all tested *Candida* isolates except CA24 that had no change in MIC values either with or without FA addition. When FLC was combined with FA, the MIC_80_ value dropped by 4–256 folds among the investigated isolates. However, CA24 showed no change in MIC_80_ of either FLC alone or in combination (Fig. 5). This confirms the potential FLC chemosensitizing activity of FA against *C. albicans* isolates.

### Fusidic acid showed negligible hemolysis on human RBCs (hRBCs)

**Fig. 6:**
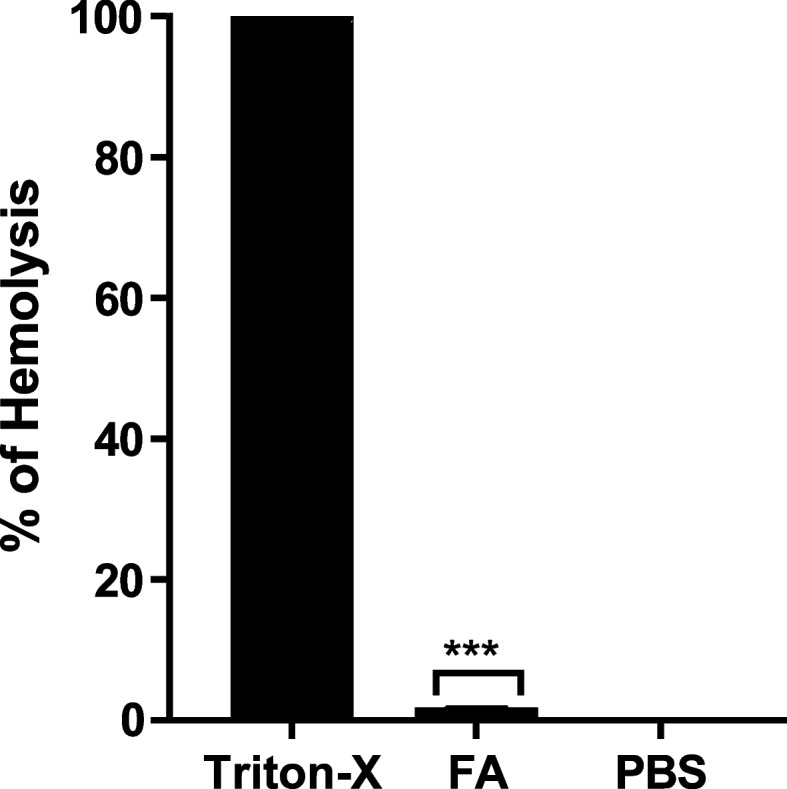
FA exhibited very low hemolytic activity to hRBCs as compared to Triton X-100 (the positive control). Data displayed was mean ± standard error and analyzed by one-way ANOVA with Bonferroni post-hoc test. ***: *P* < 0.001

Human RBCs hemolytic assay was performed to test FA toxicity. The prepared RBCs suspension was subjected to FA (1000 μg/mL). In contrast to the positive control (Triton X-100) that resulted in complete hemolysis, hRBCs were found to be minimally harmed by FA that demonstrated only 1.83% hemolysis. This finding initially suggested low toxicity of FA (Fig. 6).

### Downregulation of efflux genes by fusidic acid

**Fig. 7:**
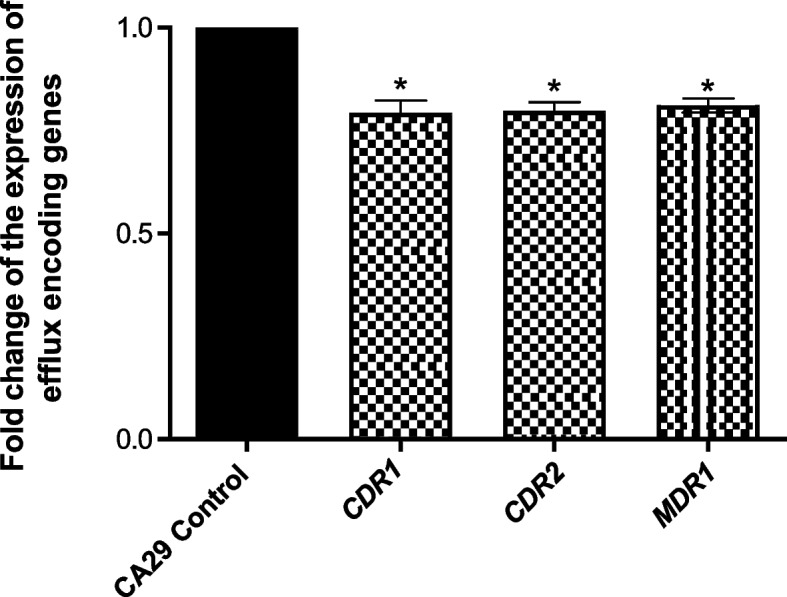
Downregulation of efflux genes by FA. A significant reduction in the expression of the *CDR1*, *CDR2*, and *MDR1* efflux genes was found in cells treated with FA. Data was analyzed using one-way ANOVA with Bonferroni post-hoc test. *: *P* < 0.05

Quantitative real-time PCR was performed to assay the inhibitory activity of FA at the molecular level (Fig. 7). The expression of *CDR1*, *CDR2*, and *MDR1* genes in *C. albicans* clinical strain CA29 treated and untreated with FA was assessed by 2^−∆∆Ct^ method. FA downregulated the expression of *CDR1, CDR2* by and *MDR1* by 20%, 17%, and 19%, respectively.

### In silico study of fusidic acid binding to *C. albicans* efflux involved proteins

**Fig. 8:**
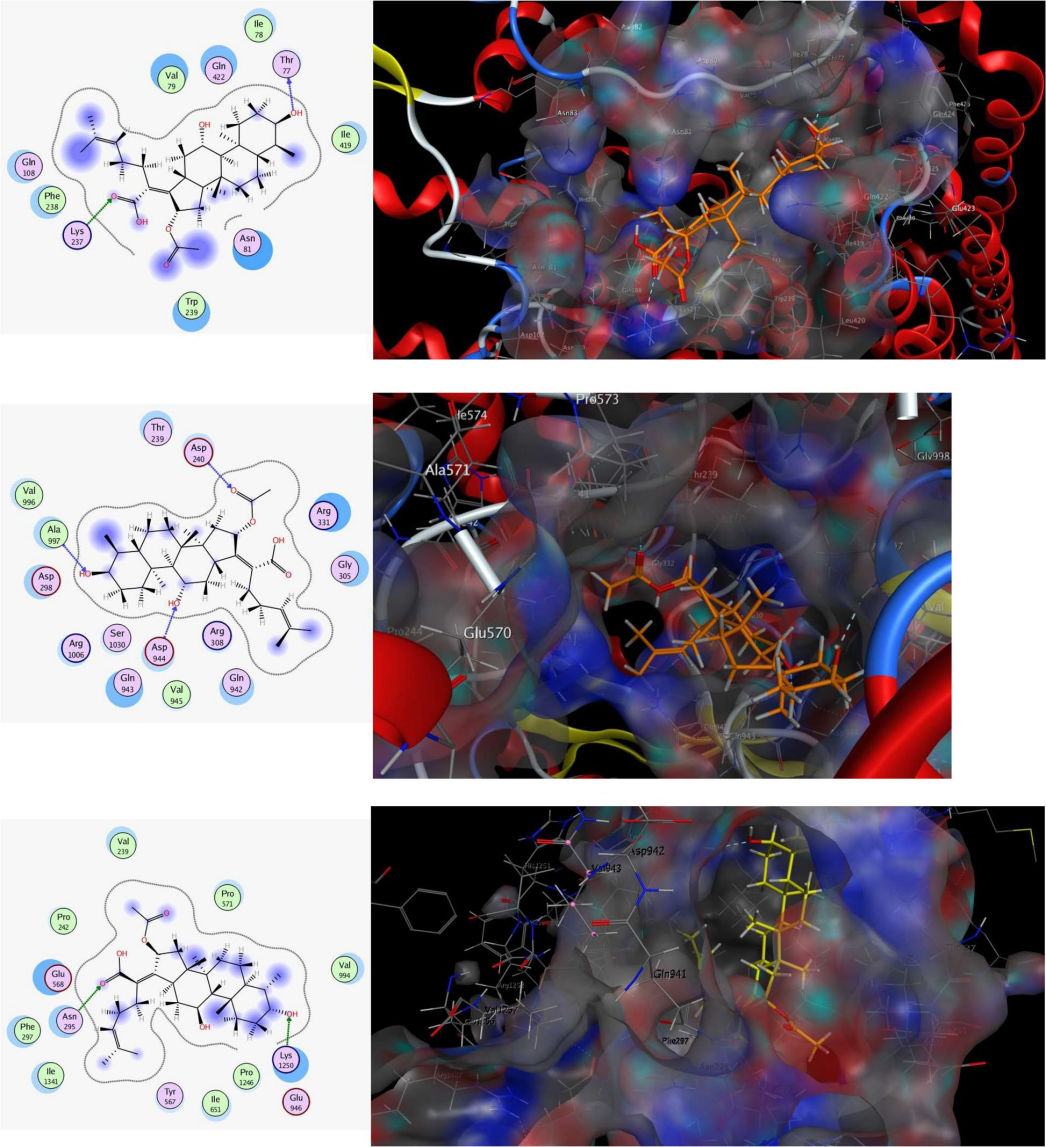
2D and 3D interactions of FA against the crystal structure of CAMdr1-AF-Q5ABU7-F1-model_v4. (top), CACdr1-AF-Q5ANA3-F1-model_v4. (middle), CACdr2-AF-P78595-F1-model_v4 (bottom)

Molecular docking is a computational technique that mimics the behavior of the molecules and molecular systems and is usually conducted to understand the binding efficiency of a ligand to its receptor. As shown in Fig. 8A, the docking results of FA against the predicted crystal structure of multidrug resistance protein 1 encoded by *MDR1* gene (AF-Q5ABU7-F1-model_v4), revealed that the ligand bound from its both extremities by two conspicuous H-bonds; the hydroxyl group at position-3 via its hydrogen atom constructed H-bond with the backbone H-bond acceptor of the conserved amino acid Thr77 while *sp2* hybridized oxygen atom attached to C-21 of the carboxylic group accepted H-bond from the H-bond donor side chain of the conserved amino acid Lys237. Besides, the hydrophobic/hydrophilic interactions elucidated from the blue shadow around the three methyl groups, one attached to C-4 and the other two attached to C-25, as well as the acetyl group (C-30, C-31) from the ligand side, and the cyan shadow around Ile78, Val79, Asn81, Gln108, Phe238, Trp239, Ile419, and Gln422 from the receptor side, improved the overall recognition and enhanced the stability of the ligand receptor complex to achieve a free binding energy -10.2714176 kcal/mol.

However, docking results against Pleiotropic ABC efflux transporter of multiple drug CDR1 protein receptor (AF-Q5ANA3-F1-model_v4) encoded by *CDR1* gene showed that the ligand was stabilized at the core of the receptor active site by three H-bonds; the hydroxyl group attached to C-3, the other hydroxyl attached to C-11, and *sp2* hybridized oxygen of the ester group attached to C-30, constructed H-bonds with the H-bond donor backbones of Ala997, Asp944, and Asp240, respectively, giving rise to score free energy of binding -11.1340609 kcal/mol (Fig. 8B).

Eventually, docking outcomes against Multidrug resistance protein CDR2 encoded by *CDR2* gene (AF-P78595-F1-model_v4) exposed that, both *sp3* hybridized oxygen of the hydroxyl group attached to C-3 and the *sp2* hybridized oxygen of the carboxylic group attached to C-21 built up two H-bonds with the H-bond donor side chain of the conserved amino acids Lys1250 and Asn295, respectively. Hence, the ligand has achieved a free binding energy of -8.97061348 kcal/mol (Fig. 8C).

Of note, H-bond acceptor and /or donor sites, which FA is rich in exhibited a persistent role in the fixation of the ligand inside the pocket of the three different receptors. Furthermore, the size and steric effect of the ligand were found to be match with the cavities of the three receptors in order of AF-Q5ANA3-F1-model_v4, AF-Q5ABU7-F1-model_v4, and then AF-P78595-F1-model_v4. So, the best inhibitory activity of FA was displayed on Pleiotropic ABC efflux transporter of multiple drug CDR1 protein receptor.

### Fusidic acid improved the antifungal effect of FLC against mice VVC

**Fig. 9:**
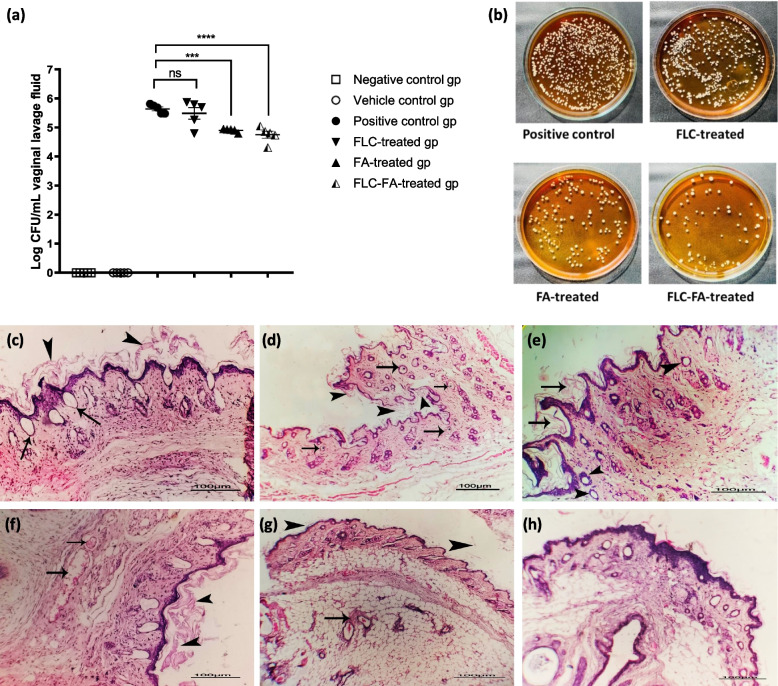
Treatment of VVC mice with FA containing gels reduced *Candida* vaginal burden after 5 days. **A** *Candida* CFU assay of vaginal lavage fluid. **B** *Candida* culture of vaginal lavage fluid on YPD agar. FLC-FA gel was the most effective treatment followed by FA gel. **C** Photomicrograph of female mice vagina 48 h post *C. albicans* infection showing presence of hyphae contact to the mucosal surface (arrows head) with cystic dilation of some glands (arrows) hyperplasia of stratified epithelium was detected without ulceration. **D** Photomicrograph of female mice vagina 5 days post *C. albicans* infection showing the presence of few hyphae contact to the mucosal surface (arrows head) with the presence of endocytosed hyphae in the mucosa (arrows). **E** Photomicrograph of female mice vagina infected with *C. albicans* and treated with FLC gel showing the presence of hyphae threads contact to the mucosal surface (arrows) with peri glandular inflammatory cells infiltration (arrows head). **F** Photomicrograph of female mice vagina infected with *C. albicans* and treated with FA gel showing the presence of hyphae threads contact to the mucosal surface (arrows head) with mild congestion of submucosal blood vessels (arrows). **G** Photomicrograph of female mice vagina infected with *C. albicans* and treated with FLC-FA gel showing minimal threads of hyphae contact to the mucosal surface (arrows head) with mild perivascular inflammatory cells infiltration (arrow) and normal mucosa. **H** Photomicrograph of female mice vagina (negative or vehicle control group) showing apparently normal mucosal and submucosal tissue with no evidence of fungal infection. Data displayed was mean ± standard error and analyzed using one-way ANOVA with Bonferroni post-hoc test. ***: *P* < 0.001, ns; non-significant. Scale bar used in histopathological analysis is 100 µm

In an immunocompromised, estrogen-dependent mouse VVC model, the efficacy of vaginal gels containing FLC, FA (1000 μg/mL), and FLC-FA [FA (1000 μg/mL)-FLC] was evaluated. Log CFU counts of vaginal lavage in treated and untreated mice were reported five days post treatment. Both FLC-FA and FA treated mice groups showed a significant decrease in *Candida* load as compared to the untreated one, although FLC-FA gel was more efficient than FA gel. There was no significant difference in *Candida* burden in the FLC-treated mice group as compared to the untreated one (Fig. 9A and B).

The mice vaginas of all groups were collected for histopathological analysis by hematoxylin and eosin staining. Two days post infection, *C. albicans* hyphae were present either in contact with the mucosal epithelium (Fig. 9C), or invaded the mucosa, destroying its structure and forming endocytosed hyphae in the deep mucosa, confirming the presence of infection. Five days post infection, hyperplasia was observed in the stratified epithelium of the infected mice vagina (Fig. 9D). Infected groups also showed a significant inflammatory response, characterized by vascular congestion, polymorphonuclear cells, and cystic dilatation of vaginal glands (Fig. 9C and D). The FLC-treated mice group demonstrated the presence of hyphae threads contact to the mucosal surface with peri glandular inflammatory cells infiltration (Fig. 9E). FA-treated mice group showed the presence of a few hyphae threads contact to the mucosal surface with mild congestion of submucosal blood vessels (Fig. 9F). FLC-FA- treated mice groups demonstrated minimal threads of hyphae contact to the mucosal surface with mild perivascular inflammatory cells infiltration and normal mucosa (Fig. 9G). The negative or vehicle control mice group exhibited a normal non-infected vagina which was confirmed by intact lamina propria with a squamous epithelial lining and mucosal glands (Fig. 9H). In conclusion, the antifungal activity of FLC against VVC in mice was enhanced by FA, therefore, FLC-FA gel was the most effective gel among the other prepared gels.

## Discussion

Resistance to antifungal drugs is a world-wide problem that affects public health. It resulted from the inappropriate use of antifungal agents in treating fungal infections. The problem of antifungal resistance represents threats such as prolonged stay in hospitals that leads to higher treatment costs. Azole drugs such as fluconazole may be ineffective against *C. albicans* due to resistance mediated by change in drug targets by mutations and the expelling of drugs outside the fungal cells by efflux pumps [50–53]. These efflux membrane proteins can expel structurally unrelated antimicrobial drugs belonging to different classes [53, 54]. Talking about azole resistance in *C. albicans*, it is principally related to the overexpression of the efflux genes *CDR1*, *CDR2* and *MDR1;* meaning that blocking these efflux pumps can lead to the reversal of azole resistance in *C. albicans* [55, 56].

Many of the antifungal medications currently available come with serious side effects, and some of them are toxic. Natural compounds that are derived from plants and different microorganisms could have the potential to be safer and more efficacious than the existing antifungals [57–60]. One of the most abundant compounds of natural origin are terpenoids and terpenes, which have a variety of bioactivities, including antimicrobial activity, antioxidant, anti-inflammatory, and antiallergic [46]. Fusidic acid is a tetracyclic triterpenoid produced by *Fusidium coccineum* that acts by inhibiting bacterial protein synthesis and is indicated for the treatment of staphylococcal infections [28]. In addition, there is no cross-resistance with the other antibiotics used in clinical practice, and Fusidic acid is hypoallergenic with low drug resistance and minimal toxicity [27, 61]. As part of a drug repurposing approach, Fusidic acid has been tested in the current study for its possible efflux pump inhibitory activity in five azole-resistant *C. albicans*. Fusidic acid has recently been reported to be a competitive inhibitor of the bile salt transporter [multidrug-resistance associated protein 2 (MRP2) and bile salt export pump (BSEP)] [62]. Both MRP2 and BSEP are ATP-binding cassette (ABC) efflux transporters and are localized mainly on the canalicular membrane of human hepatocytes [63]. The basis for the selection of fusidic acid in the study is the structural homology between ABC transporters in mammalian and fungal cells, so fusidic acid may act as a dual inhibitor of eukaryotic ABC transporters in fungal and mammalian cells. Fusidic acid was used at a concentration of 1000 μg/mL; a concentration equal to or lower than 1/16 MIC, to avoid the possible inhibitory activity of fusidic acid on the fungal growth. This was further confirmed by a viability assay with and without the tested concentration of fusidic acid. No significant difference was found between the turbidity of *C. albicans* in fusidic-treated and control untreated *C. albicans.*

Overexpression *of C. albicans* efflux pump causes fluconazole efflux to extracellular and inhibits its intracellular accumulation. As a specific substrate of ATP-binding cassette efflux pumps, rhodamine 6G was employed to assess the ability of fusidic acid on the efflux of such dye [64]. Fusidic acid was found to block the efflux of rhodamine 6G. The efflux inhibition was in the range of 54.54% to 85.84%; a finding that indicates the promising ability of fusidic acid to inhibit efflux in *C. albicans.* For further confirmation, fluorescence imaging was used to investigate the accumulation of rhodamine 6G in *C. albicans* cells either treated with fusidic acid or untreated, and treated cells showed a higher accumulation of rhodamine 6G, indicating the inhibitory activity of fusidic acid against efflux. In accordance with the current results, eucalyptal D monoterpenoid and gypenoside triterpenoid decreased the expelling of rhodamine 6G and may serve as an inhibitor of ABC efflux transporters in *C. albicans* [65]*.* Moreover, previous studies showed interference of fusidic acid with direct competitive inhibition of bile salt ABC- transporters MRP2 and BSEP [62].

 Ergosterol is one of the principal microdomain constituents within the *C. albicans* plasma membrane. Disruption of ergosterol content leads to mislocaliation of the efflux pump transporter CaCdr1p [22, 23]. Membrane transporter CaCdr1p mislocalization led to impaired function, manifested by poor efflux of azole drugs and enhanced azole activity [21]. Fusidic acid significantly reduced ergosterol content within *C. albicans* cells, which consequently could affect the localization and function of CaCdr1p. Thus, our findings suggest that Fusidic acid inhibitory activity on ergosterol could enhance its activity as an efflux pump inhibitor.

Combination therapy for the treatment of fungal infections has been the subject of extensive research in recent years. In the present study, the potentiation of the antifungal activity of fluconazole by fusidic acid was investigated. When combined with fluconazole, fusidic acid showed synergism in most tested *C. albicans* strains by 4–256 folds. Similarly, the triterpenes retigeric acid, asiatic acid, and gypenosides, greatly enhanced the activity of fluconazole against the tested *C. albicans* isolates [66–68]. Fluconazole activity against *S. cerevisiae* was also enhanced in combination with the triterpenoid capisterones A and B [69]. Similar results were obtained with several terpene and terpenoid compounds [65, 70, 71]. These results strengthen the hypothesis of the present study about the potential role of fusidic acid as a good efflux pump interfering agent.

Fusidic acid was previously reported to have minimal toxicity. To confirm the safety of fusidic acid, its effect on human RBCs was evaluated by measuring the liberated hemoglobin from lysed RBCs as a widely accepted model [72]. The possible cytotoxic activity of fusidic acid was assessed using human RBCs. Interestingly, minor hemolytic activity (1.83%) was found in RBCs treated with fusidic acid. This gave a preliminary indication of low Fusidic acid’s toxicity on mammalian cells.

For confirmation of the phenotypic results, the effect of fusidic acid on the expression of the efflux genes *CDR1, CDR2,* and *MDR1* was estimated using real-time PCR. Similarly, the use of two monoterpenes, thymol and carvacrol, at their MICs downregulated the expression of *CDR1* and *MDR1* in fluconazole-resistant *Candida* isolates [70]. However, the expression of *CDR1* and *CDR2* was increased with a sub-MIC of the diterpenoid substance oridonin compared to the untreated control *C. albicans* cells [71]. Ivanov et al., also reported that the MIC of the terpenoid compound eucalyptol upregulated the expression of *CDR1* and *CDR2*. Whereas, camphor, upregulated *CDR1* expression and downregulated *CDR2* expression [73]. The downregulation of the three genes after treatment of fungal cells with fusidic acid indicates that it is a potential efflux pump inhibitor against fluconazole-resistant *C. albicans*. Moreover, a molecular docking study was performed to investigate the possible binding of fusidic acid to the efflux transporter proteins CDR1, CDR2, and MDR1. Fusidic acid could bind to these proteins by hydrogen bonding and hydrophilic/hydrophobic interactions; a finding that gives another proof of the efflux inhibiting activity of fusidic acid. A possible explanation for the variation between phenotypic and genotypic results in the current study is that fusidic acid might have a direct interaction with the proteins, as confirmed by the molecular docking study.

Targeting fungal infections in combination therapy has little evidence of effectiveness, with mostly in vitro trials yielding controversial outcomes. Many combinations that exhibited potentiation in vitro did not demonstrate the anticipated effect in vivo [74]. Owing to these failures, other compounds, such as natural products and repurposed drugs, have been introduced to include new combinations that efficiently eradicate fungal infection, either in vivo or in vitro. To test the in vivo activity of fusidic acid to treat vaginal candidiasis, a mouse model of vaginal candidiasis that was estrogen-dependent was established. Fusidic acid was formulated as a hydrogel for topical application to overcome its low penetration and absorption through the layers of skin [75]. The mice were vaginally infected with *C. albicans* and treated with vaginal hydrogels containing fusidic acid, fluconazole, and a combination of both fusidic acid and fluconazole. A significant reduction in viable counts from vaginal lavage was found in the treated mice groups compared to the untreated group. Importantly, the group treated with fusidic acid- fluconazole combination showed the least viable counts, which refers to some kind of synergy.

As compared to the untreated infected group that showed hyperplasia in the stratified epithelium of the infected mice vaginas and a significant inflammation represented by vascular congestion, polymorphonuclear cells, and cystic dilatation of the vaginal glands, mice treated with fluconazole showed hyphae threads contact to the mucosal surface with peri glandular inflammatory cells infiltration. Moreover, mice treated with fusidic acid showed few hyphae threads attached to the mucosal surface with mild congestion of submucosal blood vessels. In the mice group treated with fusidic acid combined with fluconazole, minimal threads of hyphae attached to the mucosal surface were found with mild perivascular inflammatory cells infiltration and normal mucosa, indicating potentiation of the antifungal activity of fluconazole by fusidic acid and a promising therapeutic drug combination for vaginal candidiasis. Moreover, fusidic acid was used at a concentration of 1000 µg/mL in the current study; a much lower concentration than that indicated for the treatment of topical staphylococcal infection (20,000 µg/mL). These results are in line with Liu et al. [68], and Wang et al. [67], who found that triterpenoid compounds such as gypenosides and asiatic acid when combined with fluconazole increased the survival rate of *Galleria mellonella* larvae and reduced the invasion of the larval tissues with fluconazole-resistant *C. albicans*.

## Conclusions

The treatment of fungal infections caused by azole-resistant *C. albicans* is problematic and requires high costs. The resistance to azoles is mainly due to efflux mechanisms. Efflux pump inhibitors are thus required to reverse the resistance of *C. albicans* to azoles. Fusidic acid is a triterpenoid compound that was found to inhibit *C. albicans* efflux pumps *CDR1*, *CDR2*, and *MDR1*. Moreover, fusidic acid augmented the antifungal activity of fluconazole in vitro and in a vulvovaginitis mouse model at very low concentrations, indicating its safety for topical application that was initially demonstrated by cytotoxicity experiment on human RBCs*.* This research lays the groundwork for fusidic acids’s potential use as a clinical antifungal in the near future, adjuvant with fluconazole for azole-resistant vaginal candidiasis.

## Data Availability

All the data are presented in the manuscript.

## Abbreviations

FLC: Fluconazole
MIC: Minimal inhibitory concentration
Sub-MIC: Sub-minimal inhibitory concentration
VVC: Vulvovaginal candidiasis
R6G: Rhodamine 6G
EPI: Efflux pump inhibitors
FA: Fusidic acid
